# A network intervention that locates and intervenes with recently HIV-infected persons: The Transmission Reduction Intervention Project (TRIP)

**DOI:** 10.1038/srep38100

**Published:** 2016-12-05

**Authors:** Georgios K. Nikolopoulos, Eirini Pavlitina, Stephen Q. Muth, John Schneider, Mina Psichogiou, Leslie D. Williams, Dimitrios Paraskevis, Vana Sypsa, Gkikas Magiorkinis, Pavlo Smyrnov, Anya Korobchuk, Tetyana I. Vasylyeva, Britt Skaathun, Melpomeni Malliori, Evangelos Kafetzopoulos, Angelos Hatzakis, Samuel R. Friedman

**Affiliations:** 1 Transmission Reduction Intervention Project-Athens site, Athens, Greece; 2 Medical School, University of Cyprus, Cyprus; 3 National Development and Research Institutes, New York City, United States; 4 Quintus-ential Solutions, Colorado Springs, Colorado, United States; 5 Department of Medicine and the Chicago Center for HIV Elimination, University of Chicago, Chicago, United States; 6 Laikon General Hospital, First Department of Internal Medicine, Medical School, University of Athens, Athens, Greece; 7 Department of Hygiene, Epidemiology and Medical Statistics, Medical School, University of Athens, Athens, Greece; 8 Department of Zoology, University of Oxford, Oxford, UK; 9 Alliance for Public Health, Kyiv, Ukraine; 10 Medical School, University of Athens, Athens, Greece; 11 Organization against Drugs (OKANA), Athens, Greece

## Abstract

Early treatment, soon after infection, reduces HIV transmissions and benefits patients. The Transmission Reduction Intervention Project (TRIP) evaluated a network intervention to detect individuals recently infected (in the past 6 months). TRIP was conducted in Greece (2013–2015) and focused on drug injector networks. Based on HIV status, testing history, and the results of an assay to detect recent infections, TRIP classified drug injector “Seeds” into groups: Recent Seeds (RS), and Control Seeds with Long-term HIV infection (LCS). The network members of RS and LCS were traced for two steps. The analysis included 23 RS, 171 network members of the RS, 19 LCS, and 65 network members of the LCS. The per-seed number of recents detected in the network of RS was 5 times the number in the network of LCS (Ratio RS vs. LCS: 5.23; 95% Confidence Interval (CI): 1.54–27.61). The proportion of recents among HIV positives in the network of RS (27%) was approximately 3 times (Ratio RS vs. LCS: 3.30; 95% CI: 1.04–10.43) that in the network of LCS (8%). Strategic network tracing that starts with recently infected persons could support public health efforts to find and treat people early in their HIV infection.

Starting early antiretroviral treatment (ART) benefits HIV-infected people and reduces sexual HIV transmission^1^,^2^,^3^. Increments in HIV-RNA levels are associated with increased HIV transmission risk^4^,^5^ while substantial reductions in HIV transmission are predicted or have been observed in settings with declines in individual or community viral load^6^,^7^,^8^. Transmission during the period of acute/early infection is particularly likely^9^,^10^ because of high viral load levels^11^, lack of immune response, and elevated rates of risky behaviors^12^,^13^. Phylogenetic studies suggest that the recent phase may account for up to half of onward transmissions^13^,^14^.

Traditional contact tracing to locate and intervene with HIV-infected people is a longstanding public health strategy for identifying new cases of HIV or other Sexually Transmitted Infections^15^. Contact tracing consists of various methods to interview infected persons and elicit information about their partners in order to identify, inform, and test these contacts^15^,^16^. A review of partner counseling and referral services by health professionals has shown that up to 8% of named people who were unaware of their status tested positive for HIV^17^. Disease Intervention Specialists in the United States (US) have achieved satisfactory rates of partner elicitation and notification in New York City (NYC), and their work has resulted in substantial numbers of new HIV diagnoses^18^. However, systematic reviews have not clearly identified a single optimal strategy of partner notification^16^ and the coverage of partner notification for HIV was reportedly low^19^.

Social networks including sexual or injecting partners as well as friends and acquaintances play a role in HIV spread^15^. Studies have showed the association of network microstructures with HIV seropositivity^20^ and the role of small connected subnetworks of seronegatives in preventing HIV outbreaks^21^. A network-oriented approach in US cities identified networks in which the proportion of newly detected HIV persons was almost 5 times that of other counseling and testing contexts^22^.

We report here on an intervention using new laboratory methods to identify recently infected People who Inject Drugs (PWID) and network-based tracing to locate their network members. Our approach prioritizes recent infectees whom we hypothesize are in networks in which transmission is likely to be active. We test whether the intervention resulted in recruitment of a higher proportion of recently infected persons than a control condition that traced the networks of longer-term infected PWID.

## Methods

### Concept of the Intervention

**Figure 1:**
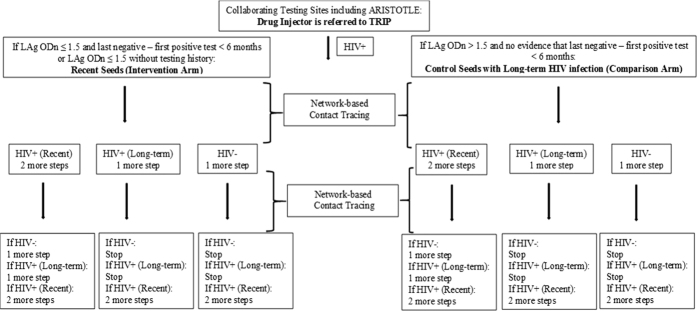
Arms and recruitment patterns of Transmission Reduction Intervention Project (TRIP). Drug injectors were referred to TRIP by collaborating testing sites. Based on the results of HIV and Limiting Antigen Avidity (LAg) testing, and on their testing history, drug injectors were grouped into the arms of the study: Recent Seeds, and Control Seeds with Long-term HIV infection. Control Seeds were matched to Recent Seeds for age (±5 years) and gender. The networks of Seeds were traced for two steps. The recruited network members underwent HIV, LAg, and viral load testing. Recents in networks were newly HIV-diagnosed network members of Seeds with documented testing history of recent infection in the last 6 months (last negative – first positive test < 6 months) irrespective of their LAg standardized Optical Density (ODn) value or had only LAg ODn ≤ 1.5 if testing history was unknown. Long-term infected participants in networks were those not classified as Recents.

The Transmission Reduction Intervention Project (TRIP) is a network-based contact tracing intervention (Fig. 1) focusing on those who are recently HIV-infected (in the past six months)^23^. If a person recruited by TRIP has recently acquired HIV, it is likely that the person who infected him/her, others whom that infector may recruit, and those whom the index person has infected are located in the same network. Recruiting and testing network members of recent infectees is thus likely to identify more people who are recently infected and probably highly infectious.

TRIP also acts to educate people about recent infection, distributes alerts within networks of recently infected persons about the presence of highly infectious individuals, and links infected participants to care^23^.

All terms and definitions used in this intervention are given in Table 1. The data set for these analyses is available in a supplementary file.

### Setting

TRIP was conducted (6/2013–7/2015) in Athens, Greece, where an HIV epidemic among PWID began in 2011^24^,^25^,^26^.

### Laboratory methods

HIV testing programs, mainly ARISTOTLE^27^, referred HIV seropositive clients to TRIP. Blood samples were tested by AxSYM HIV-1/2 gO (Abbott) and confirmed by Western Blot (MP Diagnostics). All HIV+ participants of TRIP were also tested by the Limiting Antigen Avidity (LAg) assay (Sedia^TM^ Biosciences Corporation)^28^. LAg is based on antibody maturation and categorizes HIV infection as recent versus (vs.) long-standing. The standardized Optical Density (ODn) score of 1.5 is used as cut-off for recency (130 days)^28^. HIV-RNA was quantified in all HIV positive samples in TRIP using Artus HI Virus-1 RG RT-PCR (Qiagen).

### Eligibility criteria and TRIP arms

Eligible TRIP participants were: 18 years or older; able to answer the questionnaire; and qualified for one of the project arms (Fig. 1).

The intervention arm consisted of Recent Seeds (RS) - where “Seed” refers to a primary participant recruited by TRIP - and their network members. RS were newly HIV-diagnosed drug injectors referred from ARISTOTLE or other collaborating testing facilities who had LAg ODn ≤ 1.5 and documented seroconversion in the previous 6 months or had only LAg ODn ≤ 1.5 if testing history was unknown.

The second (comparison) arm consisted of participants with a long-term HIV infection who were also used as seeds for network tracing (Control Seeds with Long-term HIV infection or LCS) and of their network members. LCS were newly HIV-diagnosed but not recently infected drug injectors who were referred from testing projects and had LAg ODn > 1.5 without any evidence of seroconversion in the last 6 months. LCS were matched to RS for age (±5 years) and gender.

### Questionnaire

All TRIP participants were interviewed by experienced personnel. The questionnaire asked the participants to name their network members: people they injected or had sex with in the past six months; people who injected or had sex in their presence in the past six months; and people who injected, used drugs or had sex with people the participants had injected or had sex with. TRIP staff also asked them to indicate venues they usually visit to use drugs, to have sex, or to meet new sex partners. Participants were asked to provide identifying information about the network contacts they named, which was electronically recorded and could help the staff validate links: names and/or nicknames and type of relationship, demographics including gender, age and nationality, physical characteristics including height, build, eyes, hair, scars, tattoos, and contact details. Participants were given coupons corresponding to people they named, which the nominees had to show to the project staff before recruitment.

### Network tracing

Network members of RS and LCS were recruited for two steps (Fig. 1). We tested network members for HIV; if they were positive, we carried out LAg tests and quantified plasma HIV-RNA. Recents in networks were defined as newly HIV-diagnosed individuals with documented testing history of recent infection (last negative – first positive test < 6 months) irrespective of their LAg ODn value or with LAg ODn ≤ 1.5 if testing history was unknown. Antibody negative samples of network members were tested for HIV-RNA in pools of 10 to identify acute infections who were classified as recents for the analyses. In order to maximize the number of potential highly infectious network members, Borderline Recents found in networks were also recruited. These were newly HIV-diagnosed individuals who did not meet the definition of recent in networks as described above. They had, however, (i) documented or very reliable self-reported testing history of HIV infection in the last 9 months (last negative – first positive test < 9 months) irrespective of their LAg ODn value or (ii) had unknown testing history but very high viral load typical of recent infection (>5 log_10_ copies/ml) regardless their LAg ODn value. For the latter case, in order to exclude long-term infected persons with Acquired Immune Deficiency Syndrome (AIDS), we evaluated clinical data on CD4 T-cell counts or diagnoses of AIDS-defining illnesses.

Long-term infected network members were those not classified as recents or borderline recents in seeds’ networks.

One person participated twice as network member of a RS and as network member of a LCS. This person was treated as a separate member of each network and both of his/her observations were included in the analysis.

If a recent or a borderline recent was found in networks of seeds, the network members of the newly identified recents/borderline recents were recruited for 2 additional steps (Fig. 1).

Unless stated otherwise, recents and borderline recents were analyzed together, and they would be referred to hereafter simply as “recents”.

### Follow-up

Follow-up interviews were scheduled at 6 months after enrollment. TRIP staff tested people who were negative at baseline for HIV to detect seroconverters. Networks of seroconverters were also traced.

### Incentives and Benefits of participation

Participants were given 10 euros for the baseline interview and 10 euros for the follow-up visit. They also received 5 euros for each named network member who participated in TRIP.

The project staff educated affected communities about recent/acute HIV infection, and about the importance of avoiding stigma. Participants were provided with standard counseling and were actively linked to care if appropriate.

### Statistical methods

Statistical analyses included chi-squared, Mann-Whitney, and Kruskal-Wallis tests.

The following metrics (Table 1) were compared between the RS and LCS groups: Network Contact Tracing Yield (NCTY) i.e. the per-seed detection of recents in networks; Proportion of Recents in Network (PRN); Proportion of Recents among Positives in Network (PRPN); and Recents Incidence in Network (RIN).

For NCTY ratios, 95% confidence intervals (CI) were constructed by treating NCTYs as incidence rates. The 95% CI of RIN ratios were constructed by treating RIN as cumulative incidence and using Poisson regression models with robust variance. The log-binomial model was used to construct 95% CI for PRN and PRPN ratios^29^,^30^.

Multivariable analyses controlled for age, gender, nationality, education, place of residence, unemployment, drug injection, and sex work.

All statistical tests were two-sided and conducted in STATA 12.

### Ethical Statement

The intervention (ClinicalTrials.gov identifier: NCT01827228) was approved by the Institutional Review Boards of the National Development and Research Institutes (NDRI) in NYC and of the Hellenic Scientific Society for the study of AIDS and Sexually Transmitted Diseases in Athens. All experiments were performed in accordance with relevant guidelines and regulations. All participants provided written informed consents.

## Results

Table 2 presents characteristics of 278 subjects including 23 Recent Seeds, 171 network members of RS, 19 Control Seeds with Long-term HIV infection, and 65 network members of LCS. The groups of seeds and their networks were generally similar. Sex work, however, was more prevalent (P = 0.051) in the networks of LCS (16.9%) than in the networks of RS (8.2%). All sex workers were drug injectors.

In total, 150 participants were HIV positive (54%) (Table 3). HIV prevalence was significantly higher in the networks of LCS (57%) than in the networks of RS (42%) and among sex workers in the networks of LCS (81.8%) than among sex workers in the networks of RS (50%).

Forty-five persons were classified as recents including 23 RS and 22 recents in the networks of seeds. The median viral load of the 45 recents was 5.4 log_10_ copies/ml (Interquartile Range (IQR): 4.6–6.0) and was significantly higher (P < 0.001) than the median viral load of all the long-standing infections in TRIP (4.7 log10 copies/ml; IQR: 2.8–5.4).

There were 19 recents in the networks of RS, including 2 acute infections detected by HIV-RNA testing, 9 newly diagnosed infections classified as recents based on documented seroconversion within the last 6 months and/or LAg ODn ≤ 1.5, and 8 borderline recents who had LAg ODn > 1.5. Of the 8 borderline recents in the network of RS, 4 had documented HIV seroconversion in the past 9 months, 2 had reliable self-reported testing history of recent infection, and 2 were newly diagnosed without any evidence of advanced disease and with very high viral load levels (>5.6 log_10_ copies/ml). The network of LCS included 1 new HIV diagnosis with LAg ODn ≤ 1.5 (recent) and 2 borderline recents who were newly diagnosed with HIV, with LAg ODn > 1.5, without late-stage disease, and with very high HIV load levels (>5.8 log_10_ copies/ml).

The per-seed number of recents detected in the network of RS was almost 5 times the number in the network of LCS (Ratio RS vs. LCS: 5.23; 95% CI: 1.54–27.61) (Table 4). The proportion of recents (PRN) in the network of RS was higher than the proportion of recents in the LCS’s network, though their ratio failed to reach statistical significance (Ratio RS vs. LCS: 2.41; 95% CI: 0.74–7.86). The proportion of recents among HIV positives in the network of RS was 27% and more than 3 times (Ratio RS vs. LCS: 3.30; 95% CI: 1.04–10.43) the proportion of recents among HIV positives in the network of LCS (8%). When borderline recents were excluded, the ratio estimate increased (5.73) but became non-significant. Similar but non-significant patterns were observed for the Recents Incidence in Network (RIN) (Ratio RS vs. LCS: 1.65; 95% CI: 0.52–5.24).

Adjusting for variables included in Table 2 did not change the ratio estimates.

## Discussion

TRIP found significantly higher yields of recents in the networks of recent seeds than in those of longer-term infected seeds on two of its measures of project effect: the yield of recents per seed, and the proportion of recents among HIV-positives in the network. On the other two measures, the proportion of recents in the networks and the incidence of recent infection in the networks, the comparison ratios ranged from 1.6 to 4.2 but the confidence intervals overlapped. Nonetheless, the overall pattern of results provides a proof of concept for the underlying hypothesis that the networks of recents will contain more recents than the networks of longer-term HIV-positives^23^,^31^.

The present findings suggest that efforts to seek, test, and treat recents can be accelerated by using strategic, network-based approaches. Finding and treating people soon after infection protects their health; and fears about toxicity and resistance have not been supported by carefully-conducted trials^2^,^3^. Further, reducing viral loads as early as possible is likely to decrease the expected number of transmissions in a community^6^,^32^,^33^. Although a mathematical model of the South African epidemic^34^ questioned whether targeting recents there would reduce transmissions over the long term, both the generalizability of the epidemic to other settings and the adequacy of the modeling have been challenged by other modelers^35^ and phylogeneticists^36^. Finally, recents and especially acutely infected people may also be appropriate targets for therapeutic vaccines, should these become available^37^,^38^.

One issue that might appear paradoxical is the higher proportion of HIV+ participants in the network of long-term infected seeds than in the network of recent seeds. We understand this finding as the logical consequence of the relatively short history of the HIV outbreak among PWID in Athens, which began only in 2011^39^. Long infection chains were established relatively rapidly and those infected at that time are now by definition long-term infected. Yet their networks have not had time to change all that much, so there will be a statistical tendency for their recruitment networks to include others who were infected at that time. The HIV epidemic slowed down before TRIP began. The recent seeds we were recruiting later at TRIP were members of recent infection chains at a time when improvements in harm reduction availability, the effects of ARISTOTLE as an intervention, drug users’ own protective actions, fluctuating behavioral risk, the firewalls network effect, and declining community viral loads due to ART use had reduced the rate of new infections^21^,^24^,^40^,^41^,^42^. Thus, although the recruitment of other recents is higher in the networks of recents for all the reasons stated here, they are likely to be in parts of the PWID community where long term HIV is less prevalent. Higher HIV prevalence in the networks of LCS could also be partially attributed to the fact that the proportions of sex workers and of HIV positives among sex workers were higher in the networks of LCS than in the networks of RS.

This work is subject to a number of limitations. First, fewer recents were recruited than had been anticipated because the outbreak in Athens had leveled off before TRIP started^40^. The TRIP intervention itself is a novel model. This plus staffing limitations may also have limited recruitment. Research is needed to learn how to recruit network/venue members more rapidly and cost effectively. Our team has begun such research in Ukraine. Secondly, given that this is a network intervention, sampling was neither random nor statistically independent. This limits the accuracy of confidence interval estimates. Thirdly, although the higher contact tracing ratio of recently infected people in networks of recent seeds probably reflects their higher density in the networks of recent seeds than in the networks of the longer-time infected seeds, we cannot rule out other explanations. Alternative explanations might include that it is a result of higher concern among members of the networks of recent seeds to protect themselves and others upon learning that there may be highly infectious people among their friends and networks; or greater attention by project staff at tracing recent seeds’ networks. Project staff made every effort to spend equal time in tracing both kinds of networks. Our finding that the proportion of recents among positives in networks is higher among members of recent seeds’ networks supports, however, the argument that the higher contact tracing rate is due to higher density of recents in the networks of recent seeds. Finally, the network size of recent seeds is bigger than the network size of long-term control seeds. By design, TRIP moves two steps away from seeds in both arms. In addition, if a recent was found in networks of seeds, contacts of the newly identified recents were also recruited for 2 rounds. Consequently, the difference in network size between recent seeds and long-term control seeds could be partly explained by the need to trace the network members of these additional recents who were found in the networks of recent seeds.

This paper provides evidence that strategic, social network tracing techniques can effectively locate people recently infected with HIV. Further research is needed to confirm the results, to find easier-to-implement ways to recruit social network members, and to improve the speed with which HIV-positives can be tested for recent infection preferably at the time and place of HIV testing. Next generation seek, test, and treat interventions could benefit from focusing on the seek component within the networks of those recently infected.

## Additional Information

**How to cite this article**: Nikolopoulos, G. K. *et al*. A network intervention that locates and intervenes with recently HIV-infected persons: The Transmission Reduction Intervention Project (TRIP). *Sci. Rep.* **6**, 38100; doi: 10.1038/srep38100 (2016).

**Publisher's note:** Springer Nature remains neutral with regard to jurisdictional claims in published maps and institutional affiliations.

## Supplementary Material

Supplementary Dataset

## Figures and Tables

**Table 1:** Definitions (listed in alphabetical order within each category).

General Definitions	
Acute infection	The initial phase of HIV infection before developing antibodies.
LAg	⦁ Limiting Antigen Avidity Assay is a blood test based on antibody maturation that helps detect people with recent HIV infection.
	⦁ If the LAg-based standardized Optical Density (ODn) score of a biological sample is ≤ 1.5, the sample has probably been collected from a person who acquired HIV in the past 6 months.
Long-term infection (Long-standing infection)	An HIV infection that occurred more than 6 months ago.
Long-term infected person	A person who acquired HIV more than 6 months ago.
Partner Notification and Counseling Services (PNCS)	A broad array of services to people diagnosed with HIV that include partner notification, prevention counseling, testing for HIV and other sexually transmitted infections, and provision of and/or linkage to medical and/or psychosocial services.
Recent infection	An HIV infection that occurred in the previous 6 months.
Recently infected person (Recent infectee)	A person who acquired HIV in the previous 6 months.
Recency	The first 6 months of HIV infection.
Social Network Contact Tracing	An extension of partner-based services that recruits network members (sexual or injecting partners, friends, acquaintances) of HIV infected people.
TRIP	⦁ Transmission Reduction Intervention Project.
	⦁ A social network contact tracing intervention that is based on recently infected HIV+ individuals.
	⦁ It is based on the concept that recruiting and testing network members of recent infectees is likely to identify more people who are recently infected or highly infectious and are thus more likely to pass the infection on to others.
	⦁ It takes actions to reduce transmission such as educating people about recent infection, distributing alerts within recents’ networks about the presence of highly-infectious individuals (targeting also the untested fraction of networks) and linking infected clients to care and antiretroviral treatment.
Operational Definitions	
Borderline Recent	⦁ A person detected in networks of Recent or Long-term Control Seeds who marginally failed to meet the operational definition of Recent in network (see below) but was treated as Recent in terms of network contact tracing (a two-step recruitment process follows).
	⦁ He/she was a newly HIV-diagnosed person with documented or very reliable self-reported testing history of infection in the last 9 months (last negative – first positive test < 9 months) irrespective of his/her LAg ODn value or he/she had unknown testing history but very high viral load typical of recent infection (>100,000 or 5 log_10_ copies/ml) irrespective of his/her LAg ODn value.
Control Seed with Long-term HIV infection (LCS)	⦁ A newly HIV-diagnosed but probably not recently infected drug injector who was referred from collaborating testing facilities and who had LAg ODn >1.5 without any evidence of seroconversion in the last 6 months.
	⦁ LCSs and their network members comprised a control arm.
	⦁ LCSs were matched to Recent Seeds for age (±5 years) and gender.
	⦁ Many LCSs first learned about their infection at around the time of their TRIP baseline interview.
	⦁ LCSs were asked to elicit names of and help recruit and test members of their social networks.
	⦁ Network recruitment for LCS stopped at second degree network members unless a Recent or a Borderline Recent was identified; then a new two-step recruitment process began.
Recent Seed (RS)	⦁ A newly HIV-diagnosed and probably recently infected drug injector referred from collaborating testing facilities who had documented seroconversion in the previous 6 months (last negative – first positive test < 6 months) and LAg ODn ≤1.5 or had only LAg ODn ≤1.5 if testing history was unknown.
	⦁ RSs and their network members comprised the Intervention Arm.
	⦁ RSs were asked to elicit names of and help recruit and test members of their networks.
	⦁ Network recruitment for RS stopped at second degree network members unless a Recent or a Borderline Recent was identified; then a new two-step recruitment process began.
Recent	⦁ A person detected in networks of Recent or Long-term Control Seeds who is probably recently infected with HIV.
	⦁ He/She is a newly HIV-diagnosed network member with documented testing history of recent infection (last negative – first positive test < 6 months) irrespective of his/her LAg ODn value or with only LAg ODn ≤1.5 if testing history was unknown.
	⦁ The term includes acutely infected persons in TRIP i.e. network members whose samples were antibody negative but tested HIV RNA positive in pools of 10.
Seed	⦁ Primary participant (drug injector) recruited by TRIP who had been tested and found HIV positive by collaborating testing and counseling facilities.
	⦁ Seeds’s specimens underwent LAg testing in TRIP.
	⦁ Seeds were asked to elicit names of and help recruit and test members of their networks.
	⦁ Network recruitment for Seeds stopped at second degree network members unless a Recent or a Borderline Recent was identified; then a new two-step recruitment process began.
Definitions of Metrics	
Network Contact Tracing Yield (NCTY)	⦁ It is calculated by dividing the number of Recents/Borderline Recents identified in Seeds’ networks by the initial number of people who were enrolled as Seeds.
	⦁ It represents the ability to detect Recents/Borderline Recents in networks of people who are used as Seeds for recruitment.
Proportion of Recents in Network (PRN)	⦁ It is calculated by dividing the number of Recents/Borderline Recents identified in Seeds’ networks by the size of these networks.
	⦁ It represents the prevalence of Recents within networks.
Proportion of Recents among Positives in Network (PRPN)	⦁ It is calculated by dividing the number of Recents/Borderline Recents identified in Seeds’ networks by the total number of HIV positives identified in these networks.
	⦁ It is the prevalence of Recents/Borderline Recents among the positives in networks of Seeds.
Recents Incidence in Network (RIN)	⦁ It is calculated by dividing the number of Recents/Borderline Recents identified in Seed’s networks by the total number of Recents and HIV negatives in these networks.
	⦁ It is a proxy for HIV incidence in networks.

**Table 2:** Characteristics of participants (n = 278) of the Transmission Reduction Intervention Project (TRIP) in Athens, Greece, 2013–2015.

	All	Recent Seeds (RS)	Network of Recent Seeds	Control Seeds with Long-term HIV infection (LCS)	Network of Control Seeds with Long-term HIV infection
Total	278	23	171	19	65
Males	219 (78.8%)	18 (78.3%)	136 (79.5%)	16 (84.2%)	49 (75.4%)
Median Age in years (Interquartile Range)	34 (30–40)	38 (30–43)	35 (30–40)	36 (32–40)	33 (30–37)
Greek national	245 (88.1%)	21 (91.3%)	150 (87.7%)	17 (89.5%)	57 (87.7%)
Permanent resident of Athens (living in Athens since birth)	145 (52.2%)	11 (47.8%)	92 (53.8%)	12 (63.2%)	30 (46.2%)
Education (up to high school)	239 (86.0%)	21 (91.3%)	147 (86.0%)	16 (84.2%)	55 (84.6%)
Homeless	76 (27.3%)	6 (26.1%)	45 (26.3%)	4 (21.1%)	21 (32.3%)
Unemployed/unable to work	233 (83.8%)	19 (82.6%)	145 (84.8%)	15 (79.0%)	54 (83.1%)
People who inject drugs (injecting over the last 6 months)	251 (90.3%)	23 (100%)	149 (87.1%)	19 (100%)	60 (92.3%)
Duration of injection	13 (7–17)	13 (3–19)	12.5 (6.5–18)	12 (7–16)	13 (7–15)
On drug/alcohol treatment at enrollment	100 (36.0%)	8 (34.8%)	64 (37.4%)	6 (31.6%)	22 (33.9%)
Heterosexuals	271 (97.5%)	22 (95.7%)	167 (97.7%)	18 (94.7%)	64 (98.5%)
Sex workers	28 (10.1%)	1 (4.4%)	14 (8.2%)	2 (10.5%)	11 (16.9%)
Male sex workers (% of males)	6 (2.7%)	1 (5.6%)	2 (1.5%)	0	3 (6.1%)
Female sex workers (% of females)	22 (37.3%)	0	12 (34.3%)	2 (66.7%)	8 (50%)

P-values for all comparisons among the four groups were > 0.05. The term network here refers to participants’ risk or social contacts who were eventually recruited and not to the entire sociometric network.

**Table 3:** HIV diagnoses and viral load level of participants (n = 278) of the Transmission Reduction Intervention Project (TRIP) in Athens, Greece, 2013–2015.

	All	Recent Seeds (RS)	Network of Recent Seeds	Control Seeds with Long-term HIV infection (LCS)	Network of Control Seeds with Long-term HIV infection
Total	278	23	171	19	65
HIV diagnoses (% of Total)	150 (54%)	23 (100%)	71 (42%)	19 (100%)	37 (57%)
Recent/Borderline Recents (% of HIV diagnoses)	45 (30%)	23 (100%)	19 (27%)	0	3 (8%)
Median HIV load for HIV positives, log_10_ copies/ml (Interquartile Range)	4.9 (3.2–5.7)	5.4 (4.6–6.0)	4.8 (3.1–5.6)	5.1 (3.9–5.7)	4.7 (1.8–5.3)
Median HIV load for Recent/Borderline Recents, log_10_ copies/ml (Interquartile Range)	5.4 (4.6–6.0)	5.4 (4.6–6.0)	5.4 (4.8–5.8)	—	5.8 (4.0–6.6)

P-value was 0.03 for the comparison of the proportions of HIV diagnoses between seeds’ networks; P-value was 0.02 for the comparison of the proportions of recents among HIV-positives between seeds’ networks. The term network here refers to participants’ risk or social contacts who were eventually recruited and not to the entire sociometric network.

**Table 4:** Yield ratios for strategic identification of recents in the Transmission Reduction Intervention Project (TRIP) in Athens, Greece, 2013–2015.

	Recents/Borderline Recents in network of Recent Seeds (RRS)	Recent Seeds (RS)	RRS/RS	Recents/Borderline Recents in network of Control Seeds with Long-term HIV infection (RLCS)	Control Seeds with Long-term HIV infection (LCS)	RLCS/LCS	Comparison for NCTY, RS vs LCS (95% Confidence Interval - CI)
Network Contact Tracing Yield (NCTY) 1 (ability to detect recents)	19	23	0.83	3	19	0.16	5.23 (1.54–27.61)
Network Contact Tracing Yield (NCTY) 2 (no borderline recents)	11	23	0.48	1	19	0.05	9.09 (1.32–391.13)
	Recents/Borderline Recents in network of Recent Seeds (RRS)	Network Size of Recent Seeds (NSRS)	RRS/NSRS	Recents/Borderline Recents in network of Controls with Long-term HIV infection (RLCS)	Network Size of Control Seeds with Long-term HIV infection (NSLCS)	RLCS/NSLCS	Comparison for PRN, RS vs LCS (95% CI)
Proportion of Recents in Network (PRN) 1 (prevalence of recents)	19	171	0.11	3	65	0.05	2.41 (0.74–7.86)
Proportion of Recents in Network (PRN) 2 (no borderline recents)	11	171	0.06	1	65	0.02	4.18 (0.55–31.74)
	Recents/Borderline Recents in network of Recent Seeds (RRS)	Positives in network of Recent Seeds (PRS)	RRS/PRS	Recents/Borderline Recents in network of Control Seeds with Long-term HIV infection (RLCS)	Positives in network of Control Seeds with Long-term HIV infection (PLCS)	RLCS/PLCS	Comparison for PRPN, RS vs LCS (95% CI)
Proportion of Recents among Positives in Network (PRPN) 1	19	71	0.27	3	37	0.08	3.30 (1.04–10.43)
Proportion of Recents among Positives in Network (PRPN) 2 (no borderline recents)	11	71	0.16	1	37	0.03	5.73 (0.77–42.71)
	Recents/Borderline Recents in network of Recent Seeds (RRS)	Recents/Borderline Recents and Negatives in network of Recent Seeds (RNRS)	RRS/RNRS	Recents/Borderline Recents in network of Controls with Long-term HIV infection (RLCS)	Recents/Borderline Recents and Negatives in network of Control Seeds with Long-term HIV infection (RNLCS)	RLCS/RNLCS	Comparison for RIN, RS vs LCS (95% CI)
Recents Incidence in Network (RIN) 1 (proxy for incidence)	19	119	0.16	3	31	0.10	1.65 (0.52–5.24)
Recents Incidence in Network (RIN) 2 (no borderline recents)	11	111	0.10	1	29	0.04	2.87 (0.38–21.52)

## References

[b1] Cohen M. S. . Prevention of HIV-1 infection with early antiretroviral therapy. N. Engl. J. Med. 365, 493–505 (2011). 21767103 10.1056/NEJMoa1105243 PMC3200068

[b2] Lundgren J. D. . Initiation of Antiretroviral Therapy in Early Asymptomatic HIV Infection. N. Engl. J. Med. 373, 795–807 (2015). 26192873 10.1056/NEJMoa1506816 PMC4569751

[b3] Danel C. . A Trial of Early Antiretrovirals and Isoniazid Preventive Therapy in Africa. N. Engl. J. Med. 373, 808–22 (2015). 26193126 10.1056/NEJMoa1507198

[b4] Quinn T. C. . Viral load and heterosexual transmission of human immunodeficiency virus type 1. Rakai Project Study Group. N. Engl. J. Med. 342, 921–9 (2000). 10738050 10.1056/NEJM200003303421303

[b5] Hughes J. P. . Determinants of per-coital-act HIV-1 infectivity among African HIV-1-serodiscordant couples. J. Infect. Dis. 205, 358–65 (2012). 22241800 10.1093/infdis/jir747 PMC3256946

[b6] Hull M. W. & Montaner J. S. G. HIV treatment as prevention: the key to an AIDS-free generation. J. food drug Anal. 21, S95–S101 (2013). 25214752 10.1016/j.jfda.2013.09.043 PMC4158850

[b7] Lingappa J. R. . Estimating the impact of plasma HIV-1 RNA reductions on heterosexual HIV-1 transmission risk. PLoS One 5, e12598 (2010). 20856886 10.1371/journal.pone.0012598 PMC2938354

[b8] Wood E. . Longitudinal community plasma HIV-1 RNA concentrations and incidence of HIV-1 among injecting drug users: prospective cohort study. BMJ 338, b1649 (2009). 19406887 10.1136/bmj.b1649 PMC2675696

[b9] Wawer M. J. . Rates of HIV-1 transmission per coital act, by stage of HIV-1 infection, in Rakai, Uganda. J. Infect. Dis. 191, 1403–9 (2005). 15809897 10.1086/429411

[b10] Hollingsworth T. D., Anderson R. M. & Fraser C. HIV-1 transmission, by stage of infection. J. Infect. Dis. 198, 687–93 (2008). 18662132 10.1086/590501

[b11] Cohen M. S., Gay C. L., Busch M. P. & Hecht F. M. The detection of acute HIV infection. J. Infect. Dis. 202 Suppl, S270–7 (2010). 20846033 10.1086/655651

[b12] Koopman J. S. . The role of early HIV infection in the spread of HIV through populations. J. Acquir. Immune Defic. Syndr. Hum. Retrovirol. 14, 249–58 (1997). 9117458 10.1097/00042560-199703010-00009

[b13] Brenner B. G. . High rates of forward transmission events after acute/early HIV-1 infection. J. Infect. Dis. 195, 951–9 (2007). 17330784 10.1086/512088

[b14] Marzel A. . HIV-1 Transmission During Recent Infection and During Treatment Interruptions as Major Drivers of New Infections in the Swiss HIV Cohort Study. Clin. Infect. Dis. 62, 115–22 (2016). 26387084 10.1093/cid/civ732

[b15] Centers for Disease Control. Recommendations for partner services programs for HIV infection, syphilis, gonorrhea, and chlamydial infection. MMWR. Recomm. reports Morb. Mortal. Wkly. report. Recomm. reports 57, 1–83 quiz CE1–4 (2008). 18987617

[b16] Ferreira A., Young T., Mathews C., Zunza M. & Low N. Strategies for partner notification for sexually transmitted infections, including HIV. Cochrane database Syst. Rev. 10, CD002843 (2013). 10.1002/14651858.CD002843.pub2 PMC7138045 24092529

[b17] Hogben M., McNally T., McPheeters M. & Hutchinson A. B. The effectiveness of HIV partner counseling and referral services in increasing identification of HIV-positive individuals a systematic review. Am. J. Prev. Med. 33, S89–100 (2007). 17675019 10.1016/j.amepre.2007.04.015

[b18] Malave M. C., Shah D., Sackoff J. E., Rubin S. & Begier E. M. Human Immunodeficiency Virus Partner Elicitation and Notification in New York City: Public Health Does It Better. Sex. Transm. Dis. 35, 869–876 (2008). 18641535 10.1097/OLQ.0b013e31817d2f82

[b19] Golden M. R., Hogben M., Potterat J. J. & Handsfield H. H. HIV partner notification in the United States: a national survey of program coverage and outcomes. Sex. Transm. Dis. 31, 709–12 (2004). 15608584 10.1097/01.olq.0000145847.65523.43

[b20] Friedman S. R. . Sociometric risk networks and risk for HIV infection. Am. J. Public Health 87, 1289–96 (1997). 9279263 10.2105/ajph.87.8.1289 PMC1381088

[b21] Friedman S. R. . Network-related mechanisms may help explain long-term HIV-1 seroprevalence levels that remain high but do not approach population-group saturation. Am. J. Epidemiol. 152, 913–22 (2000). 11092433 10.1093/aje/152.10.913

[b22] Kimbrough L. W. . Accessing Social Networks With High Rates of Undiagnosed HIV Infection: The Social Networks Demonstration Project. Am. J. Public Health 99, 1093–1099 (2009). 19372521 10.2105/AJPH.2008.139329 PMC2679789

[b23] Friedman S. R. . Socially-integrated transdisciplinary HIV prevention. AIDS Behav. 18, 1821–34 (2014). 24165983 10.1007/s10461-013-0643-5 PMC4004719

[b24] Nikolopoulos G. K. . Big Events in Greece and HIV Infection Among People Who Inject Drugs. Subst. Use Misuse 50, 825–38 (2015). 25723309 10.3109/10826084.2015.978659 PMC4498974

[b25] Nikolopoulos G. K. . National Income Inequality and Declining GDP Growth Rates Are Associated with Increases in HIV Diagnoses among People Who Inject Drugs in Europe: A Panel Data Analysis. PLoS One 10, e0122367 (2015). 25875598 10.1371/journal.pone.0122367 PMC4398461

[b26] Sypsa V. . Homelessness and Other Risk Factors for HIV Infection in the Current Outbreak Among Injection Drug Users in Athens, Greece. Am. J. Public Health 105, 196–204 (2015). 24524508 10.2105/AJPH.2013.301656 PMC4145040

[b27] Hatzakis A. . Design and baseline findings of a large-scale rapid response to an HIV outbreak in people who inject drugs in Athens, Greece: the ARISTOTLE programme. Addiction 110, 1453–67 (2015). 26032121 10.1111/add.12999 PMC4854521

[b28] Duong Y. T. . Recalibration of the limiting antigen avidity EIA to determine mean duration of recent infection in divergent HIV-1 subtypes. PLoS One 10, e0114947 (2015). 25710171 10.1371/journal.pone.0114947 PMC4339840

[b29] Barros A. J. D. & Hirakata V. N. Alternatives for logistic regression in cross-sectional studies: an empirical comparison of models that directly estimate the prevalence ratio. BMC Med. Res. Methodol. 3, 21 (2003). 14567763 10.1186/1471-2288-3-21 PMC521200

[b30] Petersen M. R. & Deddens J. A. A comparison of two methods for estimating prevalence ratios. BMC Med. Res. Methodol. 8, 9 (2008). 18307814 10.1186/1471-2288-8-9 PMC2292207

[b31] Vasylyeva T. I., Friedman S. R., Smyrnov P. & Bondarenko K. A new approach to prevent HIV transmission: Project Protect intervention for recently infected individuals. AIDS Care 27, 223–8 (2015). 25244688 10.1080/09540121.2014.947913 PMC4524562

[b32] Powers K. A. . The role of acute and early HIV infection in the spread of HIV and implications for transmission prevention strategies in Lilongwe, Malawi: a modelling study. Lancet 378, 256–68 (2011). 21684591 10.1016/S0140-6736(11)60842-8 PMC3274419

[b33] Wood E., Milloy M. J. & Montaner J. S. G. HIV treatment as prevention among injection drug users. Curr. Opin. HIV AIDS 7, 151–6 (2012). 22227587 10.1097/COH.0b013e32834f9927

[b34] Eaton J. W. & Hallett T. B. Why the proportion of transmission during early-stage HIV infection does not predict the long-term impact of treatment on HIV incidence. Proc. Natl. Acad. Sci. USA 111, 16202–7 (2014). 25313068 10.1073/pnas.1323007111 PMC4234601

[b35] Powers K. A., Kretzschmar M. E., Miller W. C. & Cohen M. S. Impact of early-stage HIV transmission on treatment as prevention. Proc. Natl. Acad. Sci. USA 111, 15867–8 (2014). 25368195 10.1073/pnas.1418496111 PMC4234559

[b36] Vasylyeva T. I., Friedman S. R. & Magiorkinis G. Prevention of early HIV transmissions might be more important in emerging or generalizing epidemics. Proc. Natl. Acad. Sci. USA 112, E1515 (2015). 25737538 10.1073/pnas.1424168112 PMC4386368

[b37] Barouch D. H. & Deeks S. G. Immunologic strategies for HIV-1 remission and eradication. Science 345, 169–74 (2014). 25013067 10.1126/science.1255512 PMC4096716

[b38] Rosenberg E. S. . Safety and immunogenicity of therapeutic DNA vaccination in individuals treated with antiretroviral therapy during acute/early HIV-1 infection. PLoS One 5, e10555 (2010). 20479938 10.1371/journal.pone.0010555 PMC2866663

[b39] Paraskevis D. . HIV-1 outbreak among injecting drug users in Greece: a preliminary report. Euro Surveill. 16 (2011). 10.2807/ese.16.36.19962-en 21924120

[b40] Paraskevis D. . Enhanced HIV-1 surveillance using molecular epidemiology to study and monitor HIV-1 outbreaks among intravenous drug users (IDUs) in Athens and Bucharest. Infect. Genet. Evol. 35, 109–121 (2015). 26247720 10.1016/j.meegid.2015.08.004

[b41] Zhang X. . Episodic HIV Risk Behavior Can Greatly Amplify HIV Prevalence and the Fraction of Transmissions from Acute HIV Infection. Stat. Commun. Infect. Dis. 4 (2012). 10.1515/1948-4690.1041 PMC3778933 24058722

[b42] Henry C. J. & Koopman J. S. Strong influence of behavioral dynamics on the ability of testing and treating HIV to stop transmission. Sci. Rep. 5, 9467 (2015). 25902018 10.1038/srep09467 PMC5386110

